# Controlled Generation of Dehydroascorbic Acid: A New Mechanistic Framework for High-Dose Vitamin C Anticancer Therapy

**DOI:** 10.3390/cells15131212

**Published:** 2026-07-03

**Authors:** Alexander V. Peskin

**Affiliations:** Centre for Redox Biology and Medicine, Department of Pathology and Biomedical Science, Mātai Hāora—University of Otago Christchurch, Christchurch 8011, New Zealand; alexander.peskin@otago.ac.nz

**Keywords:** cancer, vitamin C, dehydroascorbic acid, hydrogen peroxide, thiols, NADPH

## Abstract

This article reviews pharmacological strategies targeting key metabolic pathways in cancer cells and highlights their inherent limitations, including metabolic plasticity and lack of selectivity. It is proposed that these vulnerabilities can be addressed through a global redox-based approach using high-dose vitamin C. Evidence suggests that the anticancer activity of vitamin C is mediated by its oxidation to dehydroascorbic acid (DHA). Although DHA cannot be administered directly due to its instability, it can be generated in situ within the circulatory system. Once taken up by cancer cells, DHA perturbs multiple redox-sensitive processes, leading to depletion of NADPH and collapse of cellular redox homeostasis. We present a mechanistic framework outlining how controlled generation of DHA may enable a more robust and clinically effective anticancer strategy.

“What is now proved was once only imagined”

William Blake (The Marriage of Heaven and Hell)

## 1. Introduction

A recurring limitation of many metabolic anticancer strategies is that they focus on a single enzyme, pathway, or nutrient dependency. Although such targets are often essential under experimental conditions, tumour cells possess extensive metabolic flexibility and can compensate through alternative pathways, altered nutrient utilisation, or activation of stress-response mechanisms [1,2,3]. In addition, inhibition of a single target rarely occurs in isolation and is frequently limited by toxicity to normal tissues, preventing complete pathway suppression in patients. These considerations suggest that a more effective strategy may be to impose simultaneous pressure on multiple metabolic processes that collectively support tumour survival. Rather than targeting a single pathway, disruption of the cellular reducing environment has the potential to affect several interconnected systems, including glucose metabolism, thiol homeostasis, DNA synthesis, and methionine metabolism.

This paper reviews several metabolic strategies explored to date and proposes an alternative concept: controlled oxidation of vitamin C to dehydroascorbic acid (DHA) as a means of simultaneously perturbing multiple redox-sensitive metabolic pathways, thereby promoting collapse of tumour redox homeostasis through depletion of intracellular reducing capacity.

## 2. Glucose Metabolism

The first identified metabolic difference in cancer cells was their dependence on glucose, even in the presence of oxygen, as the main energy source, coupled with lactate production [4,5]. This phenomenon, known as the Warburg effect, has been consistently observed in cancer cells of various tissue origins [6].

Inhibition of increased glycolysis in tumours has been considered and targeted for anticancer treatment. Extensive reviews on research on inhibitors of pathways related to glucose metabolism and using the knowledge for patient treatment are available [7,8,9,10,11]. Here we discuss some of the most promising treatments as examples to estimate how progress in this field could lead to health benefits.

### 2.1. 2-Deoxyglucose

2-Deoxyglucose (2-DG) is a competitive inhibitor of glucose metabolism [12]. After uptake by cells, 2-DG is phosphorylated by hexokinase and inhibits glycolysis because unlike phosphorylated glucose it cannot be processed by phosphoglucose isomerase. Therefore, no conversion of phosphorylated glucose to phosphorylated fructose occurs, and ATP production downstream of this pathway is blocked. While 2-DG shows high efficacy in cell-based studies [13], clinical studies have not demonstrated comparable benefits. Although patient tolerance is generally good, anticancer efficacy is limited, even in combination therapies [14,15,16]. This discrepancy is largely attributed to metabolic plasticity, which enables tumour cells to bypass inhibited pathways [17,18].

### 2.2. Lonidamine

Lonidamine is a derivative of indazole-3-carboxylic acid and an inhibitor of hexokinase 2 [19], which is highly overexpressed in cancer cells [20].

It exhibits cytotoxicity to cultured cancer cells in combination with other drugs [21]. In addition to its metabolic role, hexokinase 2 may protect cells from apoptosis through interactions with other proteins and may contribute to metastasis [22]. However, clinical trials have not demonstrated improved patient survival [23]. Modified derivatives show increased cytotoxicity, possibly due to effects beyond hexokinase 2 inhibition [24].

### 2.3. 3-Bromopyruvate

3-Bromopyruvate (3-BP) is a pyruvate mimetic with promising anticancer activity [25]. In animal models, it can completely inhibit hexokinase 2 and significantly inhibit tumour growth [26].

However, its mechanism likely involves non-specific alkylation of thiol groups in glycolytic enzymes and other proteins [27,28]. As a highly reactive electrophile, 3-BP may also affect thiol-containing proteins in normal cells, limiting its clinical applicability.

## 3. Thiols

Thiol metabolism is widely considered a target for anticancer therapy due to the reliance of cancer cells on elevated antioxidant defences [29]. This vulnerability has historically been exploited by traditional medicine long ago by using arsenic for treatment [30]. Arsenic (As^3+^) binds strongly to sulfhydryl (-SH) groups. Although toxic and carcinogenic, it has shown therapeutic benefit at controlled doses.

Here we review some topics of thiol metabolism considered as the most promising for anticancer treatment.

### 3.1. GAPDH

GAPDH is an oxidant-sensitive thiol protein [31]. Although primarily involved in glycolysis, it also performs other functions [32]. A selective inhibitor koningic acid (KA) has been identified; it is a sesquiterpene lactone isolated from fungi and it directly binds to the active site of human GAPDH [33]. So far, no information on using KA in clinical trials is available. Interestingly thiol oxidation of GAPDH may act as redox switch to stimulate the oxidative pentose phosphate pathway by rerouting glucose [34]. This study highlighted that aiming at a single target even if it looks very promising may not be a winning strategy in anticancer action.

### 3.2. The Thioredoxin–Thioredoxin Reductase System

The thioredoxin–thioredoxin reductase system is recognized as a central for controlling cellular redox state via thiol-disulfide exchange and considered as a target for anticancer treatment [35,36]. Thioredoxin (Trx) is a small thiol protein, it can reduce disulfides by a thiol exchange mechanism, oxidising Trx to form an internal disulfide. Oxidised Trx is recycled to its reduced form by thioredoxin reductase (TrxR) which uses NAPDH as a source of reducing equivalents. While Trx and TrxR participate in various processes like transcription, proliferation, and apoptosis, we would like to specifically highlight that synthesis of deoxyribonucleotides depends on Trx, which makes the system vital for DNA synthesis [37]. Trx and TrxR inhibitors are cytotoxic for cancer cells in culture or in mouse models and the search for inhibitors that destabilize Trx and/or TrxR specifically in cancer cells with limited toxicity for patients is ongoing [38,39,40].

### 3.3. Peroxiredoxin

Peroxiredoxins (Prdxs) are thiol-containing proteins that cycle between oxidised and reduced forms [41]. They react with hydrogen peroxide exceptionally rapidly [42] and play critical roles in cell signalling [43,44]. Reduction of oxidised Prdxs depends mostly on Trx and in some cases on Grx [41,45]. There is a growing body of evidence that Prdxs promote tumour survival, progression, and metastasis, and are upregulated in cancers [46,47,48,49,50]. It has been reported that adenanthin, a diterpenoid isolated from plants that directly targets the conserved resolving cysteines of Prdxs, promotes differentiation of leukemic cells in culture [51]. Subsequent studies showed that the reaction of adenanthin with Prdxs lacks specificity, as it also reacts with other thiols, where selenoproteins appear to be even more favourable targets [52]. Thus, the remarkable biological effects of adenanthin may be attributed to a broader disruption of thiol metabolism in leukemic cells rather than to specific inhibition of Prdxs.

### 3.4. Glutathione

The importance of glutathione, GSH, for cancer cells’ progression, metastases, and resistance to treatment is widely appreciated [53,54,55,56]. The problem is that GSH is pivotal for normal cells [57] and selective targeting remains difficult.

## 4. Methionine

Methionine depletion has been shown to induce cell cycle arrest and apoptosis in cancer cells [58]. This phenomenon, known as methionine auxotrophy—where cancer cells are unable to proliferate in the absence of methionine while normal cells remain relatively unaffected—has been observed in many, although not all, human cancer cell lines [59,60].

Methionine dependence has also been explored therapeutically in vivo. Methionine-restricted diets have been investigated, and to further reduce plasma methionine levels recombinant methionine-γ-lyase (MGL) has been employed [61,62,63,64]. MGL catalyses the oxidative deamination of L-methionine to α-ketobutyrate, ammonia, and methanethiol. Although MGL demonstrated promising anticancer activity, clinical studies revealed significant adverse effects, including immunogenic reactions, gastrointestinal disturbances, and hyperammonaemia, which is harmful to the kidneys and liver [65,66].

One of the major biological functions of methionine is its involvement in DNA methylation. Although global hypomethylation is common in cancer cells, methionine depletion, particularly in combination with other anticancer agents, may produce undesirable epigenetic alterations that reprogram gene expression toward a more aggressive phenotype [67].

## 5. DNA Synthesis

Cell proliferation is a hallmark of tumours, and targeting processes related to DNA metabolism has been widely explored in cancer research. In attempts to eliminate cancer cells, several strategies have been pursued: (i) inhibition of DNA replication by targeting various stages and components of the replication machinery; (ii) suppression of DNA repair pathways, which are essential for maintaining genome stability and supporting survival of genetically unstable cancer cells, as well as resistance to DNA-damaging therapies; and (iii) interference with the supply of precursors required for DNA synthesis [68,69,70,71].

Many of these approaches have led to clinically effective anticancer drugs. Nevertheless, important limitations remain. Due to the genetic heterogeneity of tumours, some drugs are effective only in cancers carrying specific mutations. In addition, metabolic plasticity enables cancer cells to bypass biochemical blocks induced by therapy. Selectivity also remains a major challenge, and these therapies are often associated with toxic side effects. Furthermore, some highly effective anticancer agents may increase the risk of secondary malignancies. These limitations illustrate the need for therapeutic approaches that do not target a single component of DNA metabolism but instead simultaneously disrupt multiple metabolic pathways required for tumour proliferation, as proposed for DHA-mediated vitamin C therapy.

## 6. Vitamin C

Interest in using vitamin C for cancer patients’ treatment was inspired by Linus Pauling’s work [72]. Since then, there has been great progress in understanding vitamin C’s role in cell metabolism, deciphering pathways it is involved in and how it affects human health. Ultimately, the data obtained resulted in understanding of the necessity to consume a significant amount of vitamin C daily [73]. The current Recommended Dietary Allowance for vitamin C, which reaches up to 100 mg/day, appears increasingly at odds with the classical definition of a vitamin. Regarding the effect of vitamin C on cancer cells, numerous reports clearly showed toxicity [74,75,76]. To see anticancer vitamin C must be in high doses, 10 mM or more, which in patients cannot be achieved by oral consumption and needs injection into the blood. Nevertheless, effective treatment of cancer patients with vitamin C failed to materialize, as no clinical trials came with a positive recommendation [77,78,79].

### 6.1. What Is the Mechanism of Vitamin C Toxicity for Cancer Cells?

To display toxicity, vitamin C needs to be at high concentration. Vitamin C, ascorbic acid is a strong reducing agent. At neutral pH, ascorbic acid, AH_2_, presents mostly as the ascorbate monoanion (AH^-^) as the first dissociation constant is 4.1. In the presence of oxygen, AH^-^ donates an electron to produce an ascorbate radical, A^•−^ and superoxide, O_2_^•−^. Ascorbate radicals disproportionate to form oxidised vitamin C and dehydroascorbic acid (DHA), and superoxides disproportionate to form hydrogen peroxide, H_2_O_2_. The presence of metals of the transition group, like ferric, Fe^3+^, or copper, Cu^2+^, ions greatly facilitate the reaction. These ions are reduced by vitamin C very fast and then pass the electron onto oxygen. So, it is not surprising to see significant formation of hydrogen peroxide in cell culture medium in the presence of high concentrations of vitamin C [80,81,82,83].

Ultimately, experiments showing that catalase abolished vitamin C-induced cytotoxicity led to the widely accepted conclusion that hydrogen peroxide is the principal mediator of the anticancer effects of high-dose vitamin C [75,84,85,86]. However, several physiological factors make it difficult to reconcile this mechanism with the limited success of clinical trials. In cell culture medium, hydrogen peroxide can accumulate because its removal is relatively slow. In contrast, blood contains highly efficient antioxidant systems. Erythrocytes, which constitute nearly half of the circulating blood volume, are densely packed with peroxide-removing enzymes and rapidly eliminate hydrogen peroxide from the circulation [87,88]. In addition, transferrin restricts the availability of free iron ions required for metal-catalysed oxidation of vitamin C and subsequent hydrogen peroxide generation [83]. Finally, high-dose vitamin C is cleared from the bloodstream within hours after intravenous administration [89]. Collectively, these factors make it unlikely that vitamin C-derived hydrogen peroxide could accumulate in vivo at concentrations sufficient to exert direct cytotoxic effects on tumours.

This creates a paradox for the hydrogen peroxide hypothesis, a catch-22 situation. Generation of hydrogen peroxide appears necessary for cancer cell killing by vitamin C in vitro, yet physiological conditions make sustained exposure of tumours to cytotoxic concentrations of hydrogen peroxide highly improbable in vivo. It is therefore unsurprising that vitamin C-based anticancer therapy reached a conceptual impasse and that interest in this mechanism gradually waned. Nevertheless, occasional positive outcomes reported in clinical trials [77] suggest that an important aspect of the treatment mechanism may have been overlooked.

### 6.2. Is Vitamin C-Derived Hydrogen Peroxide Ultimately Responsible for Its Anticancer Effect?

Well-founded doubts have been raised as to whether H_2_O_2_ generated by high doses of vitamin C can fully account for its anticancer effects [90,91]. Thorough and carefully conducted studies addressing this question have not been published in peer-reviewed journal, likely because no alternative mechanism capable of explaining the observed toxicity had been proposed at the time [90]. However, another mechanism that warrants consideration is the involvement of dehydroascorbic acid (DHA), the product of two electron oxidation of vitamin C or dismutation of ascorbic radicals. H_2_O_2_ generated in medium for cultured cells in reaction of ascorbate with oxygen can itself react with ascorbate [92,93]. By removing H_2_O_2_, catalase prevents the formation of DHA and thus inhibits the toxic effect. The toxicity of high doses of ascorbate in cell culture could therefore be explained by generation of H_2_O_2_, followed by the formation of oxidized ascorbate. As explained in Section 6.1, in patients high-dose IV ascorbate on its own would not be able generate H_2_O_2_ and DHA in the blood in concentrations high enough for an anticancer effect. DHA has been shown to be toxic to cancer cells [94,95,96,97,98,99]. Within cells, DHA can be reduced back to ascorbic acid by a variety of reducing agents [100,101,102,103,104]. In solution, however, DHA is highly unstable and, in the absence of reductants, undergoes rapid decomposition [105,106,107]. Consequently, the direct clinical administration of DHA would present substantial technical challenges, as even freshly prepared DHA solutions intended for intravenous infusion would be expected to undergo significant hydrolysis.

DHA degradation generates reactive carbonyl compounds, including 3-deoxythreosone, which can induce extensive protein modification through mechanisms such as lysine crosslinking and adduct formation [108,109]. Nevertheless, as discussed below, consideration of the mechanisms and selectivity of DHA toxicity, together with the potential for DHA generation in the bloodstream, suggests that these limitations do not necessarily preclude the therapeutic exploitation of DHA-mediated toxicity in cancer treatment.

### 6.3. Mechanism of DHA Toxicity for Cancer Cells

DHA enters cells via glucose transporters (GLUTs), particularly GLUT1 and GLUT3. Expression of these transporters is increased in many, although not all, tumour types. [110]. Within a cell DHA can react with GSH, thioredoxin, glutaredoxin, and other thiols leading to their oxidation; recycling of oxidised vitamin C also can be performed by reductases of thioredoxin or cytochrome b_5_ [100,101,102,103,104,111]. These are all ultimately dependent on a supply reducing equivalents from NADPH. DHA can oxidise and inactivate thiol proteins to disulfide form unable to react with their substrates, as observed for GAPDH and Prdx2 [112].

**Figure 1:**
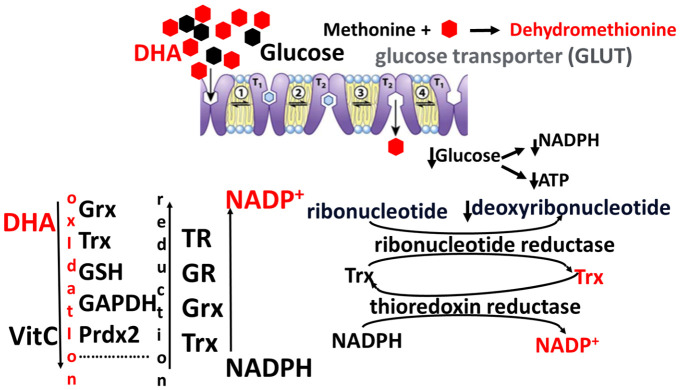
Interaction of dehydroascorbic acid (DHA) with cellular metabolism.

We can propose a complex picture of how a high concentration of DHA could exert toxicity for cancer cells (Figure 1, modified from [112]). During the uptake by a cell, high doses of DHA would compete with glucose and therefore diminish ATP production by glycolysis and NADPH when glycolysis switches to the pentose phosphate pathway. Reactions with thiols, resulting in oxidation of thioredoxin, GSH, glutaredoxin, and Prdx would put a pressure on the corresponding enzymes involved in their regeneration. These enzymes need NADPH to perform the reduction. Formation of deoxyribonucleotides needs thioredoxin and NADPH. Therefore, a deficit of precursors inflicted by DHA treatment will inevitably inhibit DNA synthesis.

DHA can oxidise methionine, potentially disrupting intracellular pathways that depend on methionine availability. DHA enters cells via glucose transporters (GLUTs) in competition with glucose. Reduced glucose uptake may diminish ATP production through glycolysis and decrease NADPH generation via the pentose phosphate pathway. Within the cell, DHA can oxidise multiple thiol groups, resulting in inhibition of thiol-dependent enzymes and increased consumption of NADPH by systems responsible for the reduction of oxidised thiols. Synthesis of deoxyribonucleotides, the precursors required for DNA replication, depends on an adequate supply of reduced thioredoxin and NADPH. Sustained intracellular accumulation of DHA may therefore impair DNA synthesis by depleting these reducing equivalents.

With prolonged DHA uptake and decreasing rate of DHA reduction in cells there is increased possibility for oxidation of thiol proteins and formation of protein adducts with DHA [103,113,114]; DHA could also interfere with S-thiolation of proteins [115]. In neuroblastoma cells, an adduct formed between DHA and homocysteine has been detected [116]. Notably, homocysteine levels are elevated in cancer patients and have been associated with tumour progression and metastasis [117].

Hydrolysis of the DHA would also result in the formation of reactive secondary products [109,110].

Additional pressure on cancer cells, which are dependent on methionine [59], can be achieved through a methionine deficit resulting from oxidation of methionine into dehydromethionine by DHA [112].

### 6.4. Selectivity of the Anticancer Effect of DHA

Could DHA bring about oxidative stress into a normal cell? Cancer cells uptake DHA preferentially whereas normal cells in culture do not show selectivity between DHA and ascorbic acid [118,119]. Therefore, in a normal cell, oxidation by DHA would be balanced by reductive power of ascorbic acid.

Reactivity of thiols with an oxidant depends on the thiol pKa, as well as the microenvironment [120,121]. While DHA effectively reacted with Prdx2 and GAPDH, it did not oxidise the thiol in p16^INK4A^ [112]. Protein p16^INK4a^ is a negative regulator of cell division, inhibiting the cyclin dependent kinases 4 and 6 and preventing progression of the cell cycle. Thiol oxidation of p16^INK4a^ leads to the formation of disulfide-bridged dimers that subsequently form amyloid fibrils. The loss of p16^INK4a^ activity is widely believed to be a common and important event in the development of cancer [122]. It is tempting to suggest that DHA may lack pro-carcinogenic action which is quite often observed for anti-cancer agents.

The DHA effect on DNA synthesis would be reversible and restricted to proliferating cells and those where NADPH is mainly provided by glucose metabolism. Notably, even with dividing cells in culture, DHA was dramatically more toxic for cancer cells than for normal ones [99].

Data on DHA toxicity available so far are restricted mostly to cells in culture. Its anticancer efficacy and lack of side effects remain to be proved in clinical trials. Thus far, no reasonable pitfalls can be seen to argue against such trials.

The mechanism of cancer cell death arising from DHA toxicity has not yet been established. Recently, a new form of programmed cell death termed disulfidptosis was described. Disulfidptosis is triggered by disulfide stress under conditions of impaired glucose metabolism and NADPH depletion, resulting in excessive S-thiolation of cytoskeletal proteins and collapse of the actin network [123,124]. Several features of DHA toxicity appear consistent with this mechanism. DHA competes with glucose for cellular uptake, oxidises intracellular thiols, and increases demand for the NADPH required for regeneration of reduced glutathione and thioredoxin. Sustained DHA exposure could therefore create conditions favouring disulfide stress. Whether DHA-induced cell death is accompanied by cytoskeletal protein S-thiolation and other hallmarks of disulfidptosis remains to be determined experimentally. If confirmed, disulfidptosis could provide a unifying mechanistic explanation linking DHA-induced oxidation of thiols, depletion of NADPH, and selective cancer cell death.

### 6.5. Does High-Dose Vitamin C Treatment Have a Future for Anti-Cancer Treatment?

High-dose vitamin C may serve as a precursor for in situ generation of DHA within the circulation, thereby exposing tumours to DHA via their blood supply. This proposal is mechanistic and exploratory rather than a defined therapeutic protocol, as the conditions required for controlled DHA generation in vivo remain to be established experimentally.

Ascorbic acid reacts efficiently with myeloperoxidase (MPO) [125], an enzyme abundant in neutrophils and monocytes that is released and activated during innate immune responses [126]. Upon activation, MPO rapidly reacts with hydrogen peroxide generated by activated leukocytes to form Compound I, which in turn oxidises ascorbic acid with a second-order rate constant of approximately 10^6^ M^−1^ s^−1^ [125].

The major physiological product of MPO is hypochlorous acid (HOCl), a potent oxidant generated by oxidation of chloride ions using hydrogen peroxide. HOCl oxidises ascorbic acid very rapidly (k = 6 × 10^6^ M^−1^ s^−1^) [127]. Although HOCl is an important component of the innate immune response [126], excessive HOCl production has been implicated in carcinogenesis and numerous pathological conditions [128,129]. We have recently found that millimolar concentrations of ascorbic acid, comparable to those achieved during high-dose intravenous vitamin C therapy, inhibit HOCl production by purified MPO through competition with chloride ions [112]. Under these conditions, ascorbic acid undergoes preferential oxidation by MPO, effectively shifting MPO activity away from HOCl production towards ascorbate oxidation [115]. Whether this can be achieved without compromising host defence or producing unintended side effects will require careful experimental validation.

**Figure 2:**
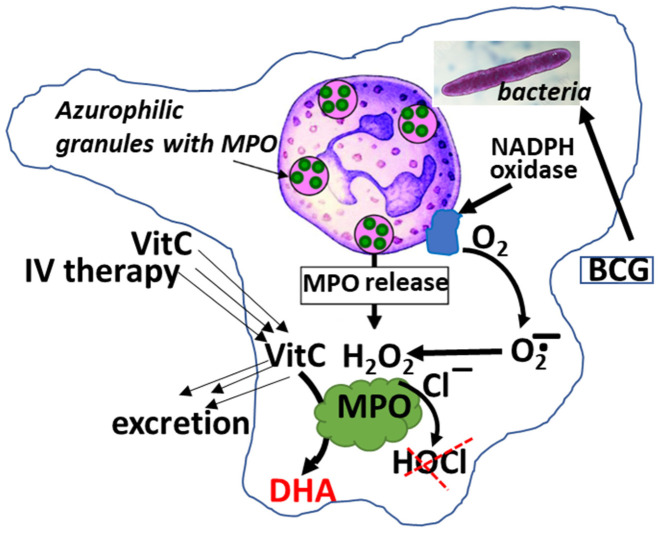
Proposed mechanism of vitamin C oxidation in blood.

MPO activity is most pronounced during innate immune responses to microbial stimuli [126]. Among clinically available options, Bacillus Calmette–Guérin (BCG), an attenuated strain of *Mycobacterium bovis* used worldwide as a vaccine and immunotherapy, represents a potential means of inducing controlled leukocyte activation and MPO release; however, its suitability, safety, and controllability in this context are entirely untested. Figure 2 depicts a hypothetical mechanism of MPO-dependent vitamin C oxidation in blood during high-dose intravenous vitamin C administration.

BCG-induced activation of neutrophils may promote hydrogen peroxide generation and MPO release. Under such conditions, elevated plasma ascorbate concentrations could potentially compete with chloride as a substrate for MPO, shifting enzymatic activity from HOCl production toward oxidation of ascorbate to dehydroascorbic acid (DHA), as observed in vitro [112]. Whether this mechanism can be reproduced in vivo in a controlled and safe manner remains unknown.

A central unanswered question is whether MPO-mediated oxidation can generate DHA at concentrations sufficient to overwhelm the reducing capacity of cancer cells. This will depend on multiple interdependent variables, including leukocyte number and activation state, MPO release kinetics, plasma ascorbate concentration, tumour perfusion, and intracellular DHA reduction and decomposition rates. At present, these parameters are not known with sufficient precision to define a dosing or timing strategy. Therefore, quantitative modelling combined with ex vivo human blood experiments will be required before any translational protocol can be considered.

### 6.6. Requirements for Successful DHA-Mediated Vitamin C Therapy

Three conditions may be necessary for successful anticancer activity:

1. Sustained millimolar concentrations of vitamin C in the circulatory system.
2. Efficient oxidation of vitamin C in blood to generate therapeutically relevant DHA concentrations.
3. Maintenance of DHA exposure for a sufficient duration to exhaust the reducing capacity of cancer cells.

High doses of vitamin C were used in clinical trials, and patient tolerance was generally good [78,79]. However, no attempts were made to induce oxidation of vitamin C in the circulation during treatment in order to ensure tumour exposure to DHA. It is possible that successful outcomes observed in some patients [79] occurred in the presence of inflammatory conditions associated with activated WBCs, resulting in MPO-mediated oxidation of vitamin C. Retrospective analysis of clinical trial data in this context may therefore be of interest, for example the blood markers of inflammation as C-reactive protein and erythrocyte sedimentation rate, which are absent in the published data.

Another limitation of vitamin C therapy was the methodology used for intravenous administration. Vitamin C is cleared from the circulation relatively rapidly, with plasma concentrations declining from millimolar to micromolar levels within a few hours after intravenous infusion [89]. Maintenance intravenous administration may therefore be required to sustain prolonged DHA generation at levels sufficient to exhaust the reducing capacity of cancer cells and achieve tumour elimination. In this regard, an analogy may be drawn with prolonged antibiotic treatment protocols.

## 7. Conclusions

High-dose vitamin C treatment may have therapeutic potential in patients with well-vascularised tumours. For successful anticancer action to occur vitamin C would need efficient oxidation in the blood. As it was discussed above, DHA can affect many targets considered attractive for anti-cancer treatment. Intracellular influx of DHA occurs in cancer cells preferentially and competes with glucose. In blood DHA can oxidise methionine and prevent cancer cells from obtaining methionine, which for them is the essential amino acid. Inside cells DHA can oxidise thiol groups resulting in overspending NADPH and over a certain period of time may result in drop of NADPH to unsustainable level. It has been suggested that maintenance of NADPH levels is often more limiting for tumour growth than are energy levels or biosynthetic precursors [130].

As there are many targets for DHA, one may suggest that metabolic plasticity of cancer cells would not be able to cover all of them. Notably, some modes of action, such as competition with glucose for cellular uptake could not be lethal on their own. Glucose concentration in blood is approximately 5 mM and therefore it would not be possible for DHA totally outcompete glucose. However, maintenance of IV vitamin C to ensure millimolar DHA levels, along with fasting regime for patients during treatment, which could bring glucose down by 20%, would contribute to the overall DHA effect by decreasing glucose influx. The therapeutic potential of methionine depletion, particularly for brain tumours where drug delivery is hindered by the blood–brain barrier, has been limited by significant adverse effects of methods used [65,66,67]. In this context, oxidation of methionine by DHA appears to be an especially attractive therapeutic strategy.

The principal strength of a DHA-based therapeutic strategy may lie in its ability to simultaneously perturb multiple metabolic processes rather than targeting a single vulnerability. Such a multifaceted mechanism could reduce the ability of cancer cells to compensate through metabolic plasticity. If efficient and controlled DHA generation in the circulatory system can be achieved safely, high-dose vitamin C may evolve from a largely empirical treatment into a rationally designed metabolic therapy. Experimental validation of this concept now represents the critical next step.

Apart from efficiency of anti-cancer treatment itself, one needs to keep in mind that patients tend to report poorer functioning and quality of life in the long term. Survivors of childhood cancer frequently develop cognitive and functional deficits that impair their ability to complete education successfully and obtain employment, ultimately reducing psychological well-being, financial security, and overall quality of life [131,132]. Currently, we are investigating how high-dose vitamin C, which is well tolerated can be effectively oxidised in human blood without side effects.

## Data Availability

No new data were created or analysed in this study.
